# Efficacy and Safety of Mirabegron Compared to Solifenacin in Treatment of Non-neurogenic Overactive Bladder in Children: A Randomized Controlled Trial

**DOI:** 10.1590/S1677-5538.IBJU.2024.0425

**Published:** 2025-01-13

**Authors:** Islam Mansour, Mahmoud Laymon, Ahmed Abdelhalim, Mohamed S. Dawaba, Ahmed S. El-Hefnawy

**Affiliations:** 1 Mansoura Urology and Nephrology Center Mansoura Egypt Mansoura Urology and Nephrology Center, Mansoura, Egypt; 2 West Virginia University Morgantown West Virginia USA West Virginia University, Morgantown, West Virginia, USA

**Keywords:** Urinary Bladder, Overactive, Therapeutics, Randomized Controlled Trial [Publication Type]

## Abstract

**Purpose:**

Non-neurogenic overactive bladder (OAB) is a common problem in children. Antimuscarinics have been widely used as first-line medical treatment. However, their frequent side effects necessitate searching for therapeutic alternatives. We aimed to assess the efficacy and safety of the beta 3 agonist, mirabegron.

**Materials and Methods:**

A randomized controlled trial enrolled child with non-neurogenic OAB refractory to behavioral urotherapy. Patients were randomized to receive either Mirabegron 25/50 mg based on a 40-kg body weight cutoff or solifenacin 5 mg for 12 weeks. Patients were assessed using Dysfunctional Voiding Scoring System questionnaire (DVSS), 3-day voiding diary and uroflowmetry. Vital signs and adverse effects were recorded at baseline and follow-up. The study primary endpoint was ≥50% reduction of the baseline DVSS.

**Results:**

Among 128 patients screened, 72 patients (36 in each group) completed the study with a mean age of 9.2±2.3 years. Both groups had significant improvement of DVSS and voiding diary (p<0.001) at 12 weeks. In mirabegron group, 94.4% (34/36) had greater than 50% improvement of DVSS compared to 75% (27/36) of solifenacin group (P=0.02). Complete symptom resolution was observed in 22.2% (8/36) patients on mirabegron versus 8.3% (3/36) on solifenacin (P=0.1). Patients on mirabegron had less adverse effects (19.4% vs 47.2%; p=0.01).

**Conclusion:**

Mirabegron is more effective with fewer adverse effects than solifenacin for treatment of children with OAB. Mirabegron treatment improves daytime symptoms and nocturnal enuresis with less risk of constipation. It may be considered as first-line pharmacotherapy in this patient population.

## INTRODUCTION

Voiding dysfunction is a common problem in the pediatric population. It affects 17-22% of children older than 5 years, the age for diagnosis (1). The term describes abnormalities of urinary bladder functions in children, either during filling or emptying (2).

Overactive bladder (OAB) is a subset of pediatric voiding dysfunction characterized by frequency, urgency, and nocturia with or without urinary incontinence in the absence of UTI or other obvious pathologies. The diagnosis relies on history taking, voiding diaries, and specific questionnaires such as the Dysfunctional Voiding Scoring System (DVSS). Evaluation of associated bowel dysfunction is important, as children with constipation are 6.8 times more likely to have voiding dysfunction (3). Clinical examination, uroflowmetry, and bladder US should be done to exclude underlying neurogenic or anatomic problems. Urodynamic studies are only considered in patients refractory to pharmacological treatment due to their invasive nature (4, 5).

The first line of treatment is behavioral urotherapy, which consists of patient education, timed voiding, proper voiding position, balanced fluid intake, and restriction of caffeine and bladder irritants. Symptoms should be evaluated after at least two months of urotherapy. For children with more severe LUTS, behavioral therapy alone has a low response and high discontinuation rate (6).

Pharmacotherapy, primarily antimuscarinics, is used as a second-line treatment for patients with OAB. However, they are frequently discontinued due to lack of efficacy or bothersome adverse effects, such as dry mouth, headache, and constipation which in turn aggravates the OAB symptoms (7, 8).

Mirabegron has been recently developed for treatment of OAB. It is a selective beta-3 adrenergic agonist that causes bladder wall relaxation. Mirabegron has shown great efficacy and safety in treating OAB in adults (8). However, a few studies have evaluated mirabegron in children, with promising results (9, 10). It was only in 2021 that the FDA approved its use in children (11, 12). We hypothesize that mirabegron is equally effective as anticholinergics in treating children with OAB refractory to behavioral therapy. The better safety profile of mirabegron can favor its use as a first-line pharmacotherapy for children with OAB,

This study aimed to assess the efficacy and safety of mirabegron for treatment of pediatric non-neurogenic OAB, compared to the antimuscarinic, solifenacin.

## MATERIALS AND METHODS

### Study design and enrollment:

**Figure 1:**
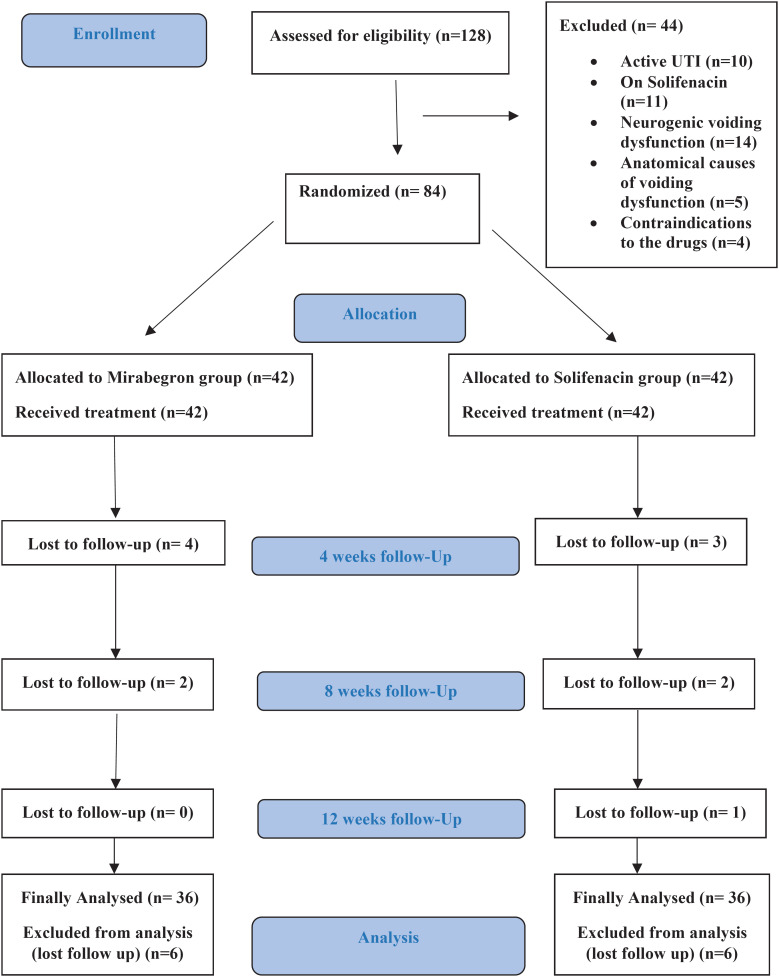
CONSORT flow chart of the progress of the parallel groups through the phases of the randomized trial.

This was a single-blinded randomized controlled trial (RCT), conducted at a single tertiary center, between February 2022 and January 2023. The study was approved by the Institutional Review Board (**MS.21.09.1680**) and registered on ClinicalTrials.gov (**NCT05240456**). Children 5-12 years of age with OAB and a DVSS score ≥ 6 for females and ≥ 9 for males, unresponsive to at least 2 months of urotherapy with or without concomitant anticholinergic treatment, were screened for eligibility (Figure-1) (13). Neurogenic or anatomical LUT abnormalities, active UTI, unresponsiveness to prior solifenacin treatment, and contraindications to solifenacin or mirabegron were the exclusion criteria. Patients who were on other anticholinergic medications at screening were instructed to discontinue anticholinergics at least 2 weeks before starting the study medication. Parents who agreed to enroll their children provided informed consent according to the Declaration of Helsinki.

### Randomization and intervention

Using the closed envelope method, patients were randomly assigned to one of the two treatment groups in a 1:1 ratio. Group 1 received 25/50 mg mirabegron orally according to their body weight. Patients <40 kg received 25 mg and patients >40 kg received 50 mg, once daily in the morning after a meal, for 12 weeks (14). Group II patients received 5 mg of oral solifenacin once daily in the morning after a meal, for 12 weeks (15). Patients were asked to fill daily dosing logs to assess compliance. Patients in both groups were asked to continue behavioral urotherapy. Constipation, if present, was concomitantly treated by increasing daily fluid and dietary fiber intake. Osmotic laxative (lactulose) was used if dietary measures were not sufficient.

### Baseline evaluation

Baseline evaluation included history and physical examination to exclude underlying neurological conditions. Heart rate and blood pressure were measured. All patients underwent urinalysis with reflex urine culture, renal bladder ultrasound, PVR measurement, and uroflowmetry. If present, UTI was treated before enrollment. Patients and their guardians were asked to complete the Arabic version of the DVSS questionnaire, a three-day voiding diary, a four-week wet night chart, and an Arabic version of the Bristol stool scale to evaluate constipation (defined as Bristol stool score of I or II) (16-18).

### Follow-up

During the 12-week study period, follow-up visits were scheduled every four weeks. During each visit, vital signs and PVR were measured. Treatment-related adverse effects were specifically questioned. Patients or their guardians were asked to refill the DVSS questionnaire. Additionally, a new uroflowmetry, three-day voiding diary, four-week wet night chart, and Bristol stool scale were obtained at the study conclusion (Table-1).

**Table 1:** Plan for treatment assessment & follow up.

Parameters	Visit 1 (evaluation)	Visit 2 (4 weeks)	Visit 3 (8 weeks)	Visit 4 (12weeks)
History taking & complete examination	✓			
Vital signs measurement	✓	✓	✓	✓
DVSS (Arabic version)	✓	✓	✓	✓
3-day voiding diary	✓			✓
Abdomino-pelvic Ultrasound	✓			
PVR	✓	✓	✓	✓
Urine analysis ± Culture	✓			
Uroflowmetry	✓			✓
Bristol stool scale	✓			✓
4-week wet night chart	✓			✓

### Endpoint

The primary endpoint was treatment efficacy, defined as ≥50% reduction of the DVSS relative to the baseline. The secondary endpoint was treatment-related adverse effects assessed at each follow-up visit. According to the American Academy of Pediatrics Clinical Practice Guidelines, clinically relevant blood pressure changes were defined as systolic blood pressure ≥140 mmHg or diastolic blood pressure ≥90 mmHg, or blood pressure more than 95th percentile for age +12 mmHg, whichever is lower (19). Clinically relevant heart rate changes were defined as ≥15 beat/minute change from baseline (20). Adverse effects were considered mild if they didn't interfere with patients’ usual functioning, moderate if they to some extent interfered, and severe if they significantly interfered.

### Sample size calculation

Assuming type I statistical error of 5% and type II statistical error of 20%, the study was powered at 80%. An average difference of 5 points in symptom score was defined as clinically relevant (21). We assumed that anticholinergic treatment would result in improved DVSS in 50% of patients. A minimum of 75% improvement of the DVSS with the new treatment was considered clinically significant, giving an effect size of 25%. With a dropout rate of 15%, a sample size of 34 patients in each study arm was estimated.

### Statistical analysis

Data were statistically analyzed using SPSS Inc., Chicago, IL, USA, version 21. Independent sample t-test, paired sample t-test, chi-square test, Mann-Whitney test, or Wilcoxon signed-rank test were used for comparisons, as appropriate. P-value ≤0.05 was considered statistically significant.

## RESULTS

### Patients

A total of 128 patients were screened for eligibility. Of them, 84 patients were included and randomized in the study (42 patients in each arm). Twelve patients did not complete the study: 2 discontinued treatment due to adverse effects and 10 lost follow-up. Therefore, the final analysis included 72 patients who completed the study (36 patients in each group). The CONSORT flow chart of the study is shown in (Figure-1). The mean age at enrollment was 9.2±2.23 years, and the mean baseline DVSS score was 15.5±3.97. A total of 27(37.5%) patients had associated constipation according to the Bristol stool scale at baseline, and 66(91.7%) patients had associated nocturnal enuresis. The baseline demographics of patients in both groups were comparable (Table-2).

**Table 2:** Baseline patient demographics.

Demographics		Mirabegron (group 1) (N=36)	Solifenacin (group 2) (N=36)	P-value
Mean age ± SD, years*		9.4 ± 2.14	9.1 ± 2.34	0.64
**Gender: N (%)**#				
	Male	14 (39%)	10 (28%)	0.32
	Female	22 (61%)	26 (72%)	
Stressful life events: N (%) #		29 (81%)	29 (81%)	1
Previous anticholinergic treatment: N (%) #		27 (75%)	25 (69%)	0.6
Mean baseline DVSS Score ± SD *		15.6 ± 4.1	15.4 ± 4	0.81
**Three-day voiding diary:**				
	Mean number of voids per day ± SD *	9 ± 2.37	9.14 ± 2.02	0.79
	Median voided volume (range), mL +	100 (30-200)	137.5 (50-300)	0.23
	Median number of daytime incontinence episodes per day, (range) +	2 (0-5)	2 (1-4)	0.34
	Nocturnal enuresis: N (%) #	33 (92%)	33 (92%)	1
	Median number of wet nights in 4-week wet night chart: (range) +	23.5 (0-28)	25 (0-28)	0.94
**Uroflowmetry:**				
	Median voided volume (range), mL +	160.5 (30-475)	138 (32-523)	0.87 0.79
	Median Q-max (range), mL/s +	21 (6-56.9)	20.9 (4.7-52.1)	
	Median PVR:(range), mL +	10 (0-60)	10 (0-50)	0.69
**Bristol stool scale: N (%)**#				
	Bristol I, II (Constipation)	14 (39%)	13 (36%)	0.97
	Bristol III, IV (normal)	21 (58%)	22 (61%)	
	Bristol V, VI (diarrhea)	1 (3%)	1 (3%)	

Comparisons done using:

* Independent sample t-test;

+ Mann-Whitney test;

# Chi square test

### Efficacy

#### DVSS

**Figure 2:**
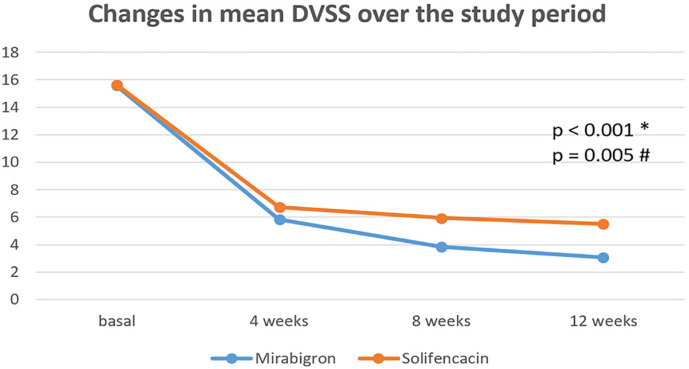
Changes in the mean DVSS scores of both groups in relation to baseline values.

At 12 weeks, the mean DVSS significantly decreased compared to baseline in both study groups (p<0.001) (Figure-2). DVSS was significantly lower in mirabegron group compared to solifenacin at 8 and 12 weeks (p=0.005).

*: Comparison of DVSS at the end of the study with baseline DVSS scores in each group.

#: Comparison of mean DVSS between both groups at the end of the study.

A total of 34 of 36 (94%) patients had ≥50% reduction of their baseline DVSS in mirabegron group compared to 27 of 36 (75%) patients in solifenacin group (p=0.02). Complete symptom resolution, a DVSS score of zero, was reported in 8(22%) patients in mirabegron group, and 3(8%) patients in solifenacin group (P=0.1).

#### Three-day voiding diary

Both groups also had significant improvement in the three-day voiding diary parameters at the end of the study compared to baseline values (P<0.001). When comparing both groups, patients on mirabegron had significantly fewer daytime incontinence episodes compared to solifenacin (p<0.001). Other variables showed no statistically significant differences (Table-3).

**Table 3:** Study outcomes.

Parameters		Mirabegron (group I) (N= 36)	Solifenacin (group II) (N= 36)	P-value
Mean DVSS score ± SD*				
	At baseline	15.6 ± 4.1	15.4 ± 4	0.81
	At 4 weeks	5.8 ± 0.52	6.7 ± 0.52	0.23
	At 8 weeks	3.8 ± 0.49	6 ± 0.54	0.005
	At 12 weeks	3.1 ± 0.51	5.5 ± 0.66	0.05
**Improvement of DVSS score: N (%)**#				
	> 50% improvement	34 (94%)	27 (75%)	0.02
	Complete symptom resolution	8 (22%)	3 (8%)	0.1
**Three-day voiding diary at 12 weeks**				
	Mean number of voids per day ± SD *	5.3 ± 1.58	6.1 ± 2.04	0.08
	Median voided volume (range), mL +	200 (50-250)	150 (85-300)	0.07
	Number of daytime incontinence episodes (range) +	(0-1)	(0-2)	<0.001
**Uroflowmetry at 12 weeks**				
	Median voided volume (mL), (range), +	177 (40-375)	149 (36-483)	0.37
	Median Q-max (mL/s), (range), +	20.7 (5.7-43.9)	23.4 (4.9-77.7)	0.96
	Median PVR at 12 weeks (range), mL +	6.5 (0-50)	6 (0-50)	0.73
**Four-week wet night chart at 12 weeks**				
	Median number of wet nights: N (range) +	6 (0-28)	6 (0-28)	0.81
	Improvement > 50%: N (%) #	22 (67%)	23 (69%)	0.79
	Complete dryness: N (%) #	5 (15%)	7 (21%)	0.53
**Bristol stool scale**				
	Bristol I, II (constipation): N (%) #	7 (19.4%)	15 (41.7%)	0.04
**Treatment related adverse effects**				
	Total Number (%) #	7 (19.4%)	17 (47.2%)	0.01
	Constipation	1 (2.8%)	6 (16.7%)	
	Headache/drowsiness	3 (8.3%)	6 (16.7%)	
	Dry mouth	0	2 (5.6%)	
	Blurring of vision	0	1 (2.8%)	
	Abdominal pain	1 (2.8%)	1 (2.8%)	
	Acne like rash	1 (2.8%)	0	
	Sweating	0	1 (2.8%)	
	Behavioral changes (Hallucination)	1 (2.8%)	0	

DVSS = Dysfunctional Voiding Symptom Score, PVR = postvoid residual. Comparisons were made using:

* independent sample t-test,

+ Mann-Whitney test,

# Chi-square test.

Significant differences are in bold.

#### Uroflowmetry

In mirabegron group, the median voided volume increased from 160.5(30-475) mL at baseline to 177(40-375) mL at 12 weeks (p=0.27). In solifenacin group, it increased from 138(32-523) mL at baseline to 149(36-483) mL at 12 weeks (p=0.39). These differences were not statistically significant (Table-3).

#### Four-week wet night chart

In mirabegron group, the number of wet nights per 4 weeks improved from a baseline median of 23.5(0-28) to 6(0-28), (p<0.001). A greater than 50% reduction in the number of wet nights was achieved in 22 of 33(67%) patients who had associated nocturnal enuresis. Complete nighttime dryness was achieved in 5 of 33 patients (15%). While in solifenacin, the number of wet nights improved from baseline median 25(0-28) to 6(0-28), (p<0.001). Improvement ≥50% was achieved in 23 of 33(69%) patients who had nocturnal enuresis. Complete nighttime dryness was achieved in 7 of 33(21%) patients.

#### Bristol stool scale

At the end of treatment, mirabegron group had significantly fewer patients suffering constipation, defined as Bristol I or II, compared to solifenacin (P=0.04) (Table-3).

#### Safety

No clinically significant blood pressure or heart rate changes were observed in both groups. Only one patient discontinued mirabegron due to chest pain, which was reversible after treatment discontinuation. Also, one patient discontinued solifenacin due to an extensive skin rash. Mirabegron showed a significantly better safety profile. Side effects were reported in 7 of 36 (19.4%) children on mirabegron, with headache (n=3) being the commonest. While 17 of 36 (47.2%) children on solifenacin had side effects of which constipation and headache were the commonest (n=6 each) **(P=0.01)**. All reported side effects were mild and fully reversible after treatment discontinuation (Table-3).

## DISCUSSION

Anticholinergic drugs have been widely used as primary pharmacological agents for children with OAB refractory to behavioral urotherapy. Solifenacin, an M3 selective antimuscarinic, has proven superior efficacy and safety compared to the traditional anti-muscarinic drugs (22-24). In this RCT, both mirabegron and solifenacin were equally effective in reducing daytime frequency, nocturnal enuresis, and increasing the median voided volumes. Notably, patients treated with mirabegron had lower DVSS and fewer daytime incontinence episodes on the 3-day voiding diary compared to those treated with solifenacin. A limited number of prospective studies have compared the efficacy of both drugs in children with OAB. A placebo-controlled RCT compared the efficacy and safety of mirabegron and solifenacin in children with newly diagnosed OAB. Based on the 3-day voiding diary for symptom evaluation, the authors reported comparable efficacy of mirabegron and solifenacin (25). Two other studies showed improved bladder capacity and daytime continence with mirabegron in children with OAB refractory to anticholinergics (9, 10).

To our knowledge, this is the first prospective trial to evaluate mirabegron efficacy in treatment of nocturnal enuresis associated with OAB using the standard 4-week wet night charts. Both groups had significant and comparable improvement in the median number of wet nights. Improvement of ≥50% of wet nights post-treatment was achieved in 67% of patients on mirabegron versus 69% on solifenacin. Two retrospective studies reported improved nocturnal enuresis in children treated with mirabegron. In one study, improvement >50% was achieved in 87.5% vs. 63.2% of patients using mirabegron and solifenacin respectively (26). In the other study, 35% of patients on mirabegron showed improvement >50% after 6 months of follow-up (27).

An overwhelming majority of 85.7% of patients assigned to mirabegron in this study were compliant to their treatment. Only one of 42(2.4%) patients discontinued mirabegron due to chest pain that resolved after treatment discontinuation. Unfortunately, that patient was lost to follow-up and the cause of his chest pain could not be investigated. This is consistent with a recent meta-analysis that reported a high likelihood of drug adherence in >80% (12). Cardiovascular adverse effects are well known with mirabegron and are a common cause for treatment discontinuation. Palpitation was reported in 8 of 279 (2.9%) adult patients who received mirabegron for OAB, 3 of those patients (1%) had chest pain. Chest pain and palpitations resolved once therapy was stopped (28). Chest pain was also reported in 1 of 41 children in a recent retrospective study of mirabegron (29). Cardiovascular side effects, like hypertension and prolonged QT interval on ECG are one of the main concerns with mirabegron treatment in adults (30). Although the same concern was raised in the pediatric population, clinically significant cardiovascular side effects were uncommon in a recent meta-analysis in children (12). The lower incidence may be explained by careful selection of cases and exclusion of patients with cardiovascular risk factors, which are uncommon in children, unlike adult patients.

Overall, adverse effects were less common with mirabegron compared to solifenacin in the current study. The most common side effects with mirabegron were headache or drowsiness in 3 (8.3%) patients, constipation, abdominal pain, acne-like rash, and behavioral changes or hallucination were reported in one patient (2.8%) each. These results were in line with the most recent studies on mirabegron safety (9, 12, 25). On the other hand, the most common side effects with solifenacin were constipation, headache or drowsiness in 6 (16.7%) patients each. Other side effects included dry mouth in 2 (5.6%) patients, visual blurring, sweating, and abdominal pain in one patient (2.8%) each. One patient (2.8%) discontinued solifenacin due to a significant skin rash. These adverse effects are also in agreement with the available literature (17, 19).

This study is limited by the relatively small sample size and the short treatment duration. The lack of external funding for the study limited the ability to enroll more patients and extend the study duration beyond 12 weeks. This study also lacked an evaluation of the treatment effect on the patients’ quality of life. The drop-out cases could not be tracked to identify drop-out causes. Patients treated with mirabegron were not evaluated with ECG before or after treatment to evaluate prolonged Q-T interval. Despite the superior efficacy and safety of mirabegron, treatment costs may limit its use as a first-line treatment in children with OAB. We look forward to future studies with longer follow-ups to demonstrate the durability of treatment effects and evaluate long-term safety and patient compliance.

Despite these limitations, to our knowledge, this is the first randomized controlled trial to demonstrate that mirabegron is more effective with fewer adverse effects than solifenacin in children with OAB. Another important advantage of this study is the use of multiple tools to assess patient symptoms including the DVSS, 3-day voiding diary, and 4-week wet night chart. The combined use of these tools permitted the concurrent evaluation of nocturnal enuresis alongside daytime symptoms and assessment of both storage and voiding symptoms. Further, the Bristol stool scale demonstrated a lower risk of constipation with mirabegron. This could have contributed to the better symptom improvement seen with mirabegron. This multi-parametric evaluation fits the complex and multi-faceted nature of voiding dysfunction in children.

## CONCLUSION

Mirabegron is more effective with fewer treatment-related adverse effects compared to solifenacin in children with OAB refractory to behavioral therapy and other anticholinergic medications. Mirabegron treatment improves daytime symptoms and nocturnal enuresis with less risk of constipation. It may be considered as a first-line pharmacotherapy for select patients with non-neurogenic OAB.

## ABBREVIATIONS

BMI: = Body mass index
DVSS: = Dysfunctional voiding scoring system
ECG: = Echocardiogram
FDA: = Food and Drug Administration
LUT: = Lower urinary tract
LUTS: = Lower urinary tract symptoms
OAB: = Overactive bladder
US: = Ultrasonography
PVR: = Post voiding residual
RCT: = Randomized controlled trial
UTI: = Urinary tract infection
